# An approach to trial design and analysis in the era of non-proportional hazards of the treatment effect

**DOI:** 10.1186/1745-6215-15-314

**Published:** 2014-08-07

**Authors:** Patrick Royston, Mahesh KB Parmar

**Affiliations:** MRC Clinical Trials Unit at UCL, Aviation House, 125 Kingsway, London, WC2B 6NH UK

**Keywords:** Time-to-event data, Randomized controlled trials, Hazard ratio, Non-proportional hazards, Logrank test, Grambsch-Therneau test, Flexible parametric model

## Abstract

**Background:**

Most randomized controlled trials with a time-to-event outcome are designed and analysed under the proportional hazards assumption, with a target hazard ratio for the treatment effect in mind. However, the hazards may be non-proportional. We address how to design a trial under such conditions, and how to analyse the results.

**Methods:**

We propose to extend the usual approach, a logrank test, to also include the Grambsch-Therneau test of proportional hazards. We test the resulting composite null hypothesis using a joint test for the hazard ratio and for time-dependent behaviour of the hazard ratio. We compute the power and sample size for the logrank test under proportional hazards, and from that we compute the power of the joint test. For the estimation of relevant quantities from the trial data, various models could be used; we advocate adopting a pre-specified flexible parametric survival model that supports time-dependent behaviour of the hazard ratio.

**Results:**

We present the mathematics for calculating the power and sample size for the joint test. We illustrate the methodology in real data from two randomized trials, one in ovarian cancer and the other in treating cellulitis. We show selected estimates and their uncertainty derived from the advocated flexible parametric model. We demonstrate in a small simulation study that when a treatment effect either increases or decreases over time, the joint test can outperform the logrank test in the presence of both patterns of non-proportional hazards.

**Conclusions:**

Those designing and analysing trials in the era of non-proportional hazards need to acknowledge that a more complex type of treatment effect is becoming more common. Our method for the design of the trial retains the tools familiar in the standard methodology based on the logrank test, and extends it to incorporate a joint test of the null hypothesis with power against non-proportional hazards. For the analysis of trial data, we propose the use of a pre-specified flexible parametric model that can represent a time-dependent hazard ratio if one is present.

## Background

Most randomized controlled trials (RCTs) with a time-to-event outcome are designed and analysed under the proportional hazards assumption, with a target hazard ratio (HR) for the treatment effect in mind. However, the hazards may be non-proportional (non-PH). If so, questions arise as to how best to design the trial and analyse the results.

We believe that there may be two reasons that non-proportional hazards are being detected more frequently nowadays. First, phase III trials are generally much larger today, giving more power in any given situation to detect non-proportional hazards. Second, with the biological revolution there are many new therapies being evaluated, having different modes of action. For example, monoclonal antibodies have been evaluated for treating many different types of cancer. They are given for a defined period of time (often one or two years) and then stopped. It is entirely plausible that the effect of the intervention might persist during the treatment period but then diminish gradually afterwards. Such behaviour, which in fact is what has been seen in the examples given above, would lead to non-proportional hazards.

Typically, the sample size calculation for such a trial utilizes some form of logrank test. An estimate of the HR with a confidence interval (CI) is often obtained from a Cox model with treatment as the only covariate. If non-PH is present, the HR is actually time-dependent and the estimated HR that is obtained is some type of average over the event times [1].

An alternative design approach is to alter the test used to analyse the data by adding a test for a possible time-dependent treatment effect. Such a joint test may have more power than the logrank test when certain patterns of non-PH are present. To obtain the same power as for the logrank test under PH, an increase in the sample size is needed, or the target power under PH may be reduced, or the significance level of the test may be relaxed. We call a test of this type a ‘joint test’. We envisage a scenario in which we pre-specify a more robust test that has power in the presence of non-PH. Once we have identified a ‘signal’, we can try to better understand its nature by further analysis of the data, as outlined in principle in the next paragraph.

Allied to the joint test, we propose the use of a joint model, which also incorporates a possible time-dependent treatment effect. Presentation of results of a joint model may include Kaplan-Meier survival curves and a pre-specified model-based estimate of the treatment effect. This may be the estimate of the HR (with CI) at particular time points, or indeed, because of the time-dependent nature of the results, other potentially more informative and clinically relevant quantities, such as differences in survival curves and in restricted mean survival times between arms [2].

The structure of the paper is as follows. In the Methods section, we motivate and describe the joint test of the HR and non-PH that we propose. We show how to re-power the logrank test under PH to achieve the power desired for the joint test. We discuss the analysis of trial data and make a case for estimating quantities of interest, particularly those with a time-dependent perspective, using a flexible parametric survival model as the joint model. We deal with the important issue of pre-specifying the analysis in the protocol and statistical analysis plan. In the Results section, we give some numerical examples showing the implications of designs incorporating the joint test for the sample size and number of events. We then present a small simulation study of the power of the joint test when non-PH is present. We finish with a discussion and conclusions.

## Methods

### Motivation

The basic issue that concerns us is that a clinically important treatment effect may be more complex than implied by a simple hazard ratio (HR) between treatment arms. For example, survival curves that diverge then converge or even cross may suggest the mode of action of a research treatment. A simple HR would not do justice to interpreting such data. One might say, ‘just plan the sample size according to the logrank test as usual, then test for non-PH after that’. But if the test of non-PH is positive, how does one proceed - particularly in cases where the logrank test does not exhibit conventional levels of significance and the non-PH test does? It should be noted that the significance level of such a sequential procedure is approximately double the nominal level of the logrank test, since two independent tests have been carried out. Doubling the probability of a type 1 error is not likely to be acceptable to trialists.

An obvious difficulty here is the very general nature of possible departures from PH. This makes it nigh impossible to confidently and convincingly pre-specify a time-dependent pattern of HR behaviour with which to power the logrank test. We believe it is preferable and practically more appealing to power it under PH, as a rough approximation to what might be expected, but with an adjustment to allow a ‘second degree of freedom’ to incorporate a rather general test of non-PH. For the latter, we suggest the Grambsch-Therneau test [3] based on the correlation between scaled Schoenfeld residuals and (our preference) the ranks of the failure times.

We assume that the Grambsch-Therneau test statistic is independent of the Cox or logrank test of the treatment effect and under PH has a central chi-square distribution on 1 degree of freedom (d.f.). (See further comments on this assumption in the next paragraph.) The sum of the logrank or Cox and Grambsch-Therneau chi-square statistics provides a test statistic whose distribution under the global null hypothesis of identical survival functions across treatment arms is central chi-square on 2 d.f.

To check the above assumptions, we ran some simple simulations which we do not report in detail. In essence, with sets of 5,000 replicated two-arm ‘trials’ with staggered patient entry, we found that, in the null case, the Grambsch-Therneau test statistic was very close to *χ*^2^ on 1 d.f. and the joint test statistic was very close to *χ*^2^ on 2 d.f. The Spearman rank correlation between the two component test statistics was close to zero. We conclude that the assumptions are sound.

### A joint test

A ‘joint test’ is a single statistic for simultaneous testing of more than one aspect of the global null hypothesis that there is no difference between survival distributions in treatment arms. The global null hypothesis implies that the HR for the treatment effect equals 1 and that the HR is independent of time (that is, PH holds). The aims of a joint test are threefold:

1. To widen the concept of ‘treatment effect’ to include the important possibility that the HR may depend on time since randomization, and in some cases, the estimated HR is ‘not significant’;
2. To have planned power to detect a treatment effect (in terms of the HR) of pre-specified magnitude under PH;
3. To have power in cases of non-PH in which the power of the logrank test alone could be compromised.

Our suggested joint test is as described above. Of course, other choices are possible, notably, based on a flexible parametric survival model with a time-dependent treatment effect [4, 5]. We comment further on this issue in the Discussion.

#### Power

We now consider the implications for sample size and power of replacing the logrank test with a joint test as just described. Suppose that there is a non-null treatment effect (HR ≠1) and PH holds. In such cases, the second component of the joint test is null, but not the first. By applying the joint test under PH, we ‘waste’ one d.f., reducing the power compared with the more parsimonious and (under PH) universally most powerful logrank test. Since the logrank test is asymptotically equivalent to a Cox test, we can equally well substitute the likelihood ratio or Wald chi-square statistic from Cox regression on the treatment indicator variable for the logrank chi-square statistic.

Let *α* be the two-sided significance level and *ω* the power of the logrank test under PH. Define


where *Φ*^-1^(.) is the inverse standard normal distribution function. When HR ≠1, the logrank chi-square statistic has a non-central *χ*^2^ distribution with 1 d.f. and non-centrality parameter *zz*^2^. The distribution of the joint test statistic under PH is non-central *χ*^2^ with the same non-centrality parameter of *zz*^2^, but with 2 d.f. instead of 1 d.f. With this setup, the power *ω*_*JT*_ of the joint test under PH is
1

where *F*_*ν*_(*λ*,*u*) is the distribution function of a non-central *χ*^2^ variate *u* with *ν* d.f. and non-centrality parameter *λ*, and  is the central chi-square deviate on *ν* d.f. corresponding to probability *p*. For example, . The required functions are widely available in software packages. In Stata, for instance, *F*_*ν*_(*λ*,*u*) and  are implemented as functions nchi2(*ν*,*λ*,*u*) and invchi2(*ν*,*p*), respectively.

We describe our preferred approach to powering the joint test in the Discussion. First, we describe three possible strategies.

#### Strategy 1: Power the logrank test as usual

This is the simplest approach. We power the logrank test as usual and use Equation (1) to calculate the resulting joint test power, *ω*_*JT*_. For example, with significance level *α*=0.05 and power *ω*=(0.8,0.9) we find *ω*_*JT*_=(0.709,0.835). Many people would probably be happy with (*ω*,*ω*_*JT*_)=(0.9,0.835), whereas they might find *ω*_*JT*_=0.709 too low for comfort.

#### Strategy 2: Power the joint test

We might prefer to specify a power *ω*_*JT*_ for the joint test at significance level *α* under PH. What corresponding value of *ω* is needed for the logrank test?

Equation (1) can be rearranged to determine *ω* given *ω*_*JT*_:
2

where *G*_*ν*_(*u*,*p*) is the non-centrality parameter of a non-central *χ*^2^ variate with *ν* d.f. at a value *p* of the distribution function. If *F*_*ν*_(*λ*,*u*)=*p*, then *G*_*ν*_(*u*,*p*)=*λ*. In Stata, *G*_*ν*_(*u*,*p*) is implemented as function npnchi2(*ν*,*u*,*p*).

For example, suppose we require *ω*_*JT*_=0.8 at *α*=0.05. According to Equation (2) we find that , . The study would therefore need power 0.874 for the logrank test under PH, with a corresponding increase in sample size.

#### Strategy 3: Relax the significance level of the joint test

An alternative to increasing the power of the logrank test, and hence the sample size, is to increase the significance level of the joint test to achieve power *ω* for the joint test. We wish to determine the two-sided significance level, *α*_*JT*_, for the joint test to have power *ω* under PH. With this setup, the power of the joint test is


where *zz*=*z*_1-*α*/2_+*z*_*ω*_ as before. Inverting this expression, we obtain


where, for instance,  is implemented in Stata as invnchi2(*ν*,*λ*,*p*). For example, with *α*=0.05, *ω*=0.9 we find *α*_*JT*_=0.0985 (that is, nearly 2*α*). With *α*=0.05, *ω*=0.8, then *α*_*JT*_=0.0952. Taking *α*_*JT*_≃2*α* is likely to be adequate in practice for 0.8≤*ω*≤0.95. In most trials, power is set at 0.8 or 0.9.

### Presentation of results

Traditionally, studies with a time-to-event outcome are designed according to a target HR and reported with an estimate of the resulting HR and a 95*%* confidence interval, usually obtained from a Cox regression as already mentioned. The HR may or may not be adjusted for covariates such as important prognostic factors or stratifying variables. Often, a graph of the Kaplan-Meier estimates of the survival curves is presented.

In the emerging era in which the PH assumption may fail [6, 7] (for reasons we could speculate about), the traditional strategy seems inadequate. We have argued [2] for estimating and presenting alternative measures of a treatment effect which do not rely on a time-independent HR. Here we suggest producing two closely related reports: graphical and quantitative.

#### Graphical presentation

The key understanding is that most measures of clinical and statistical relevance in the non-PH context are *time-dependent*. These include, obviously, the traditional survival functions, but also the HR, the difference in restricted mean survival time between treatments [2], and the difference in survival functions between treatments. We propose presenting plots of all four of these quantities against time in a single graphic image.

Restricted mean survival time (RMST) for a mortality outcome in a trial may loosely be described as the life expectancy over the restricted period between randomization and a defined, clinically relevant time horizon, usually called *t*^∗^. With uncensored survival times *t*_1_,…,*t*_*n*_ we would first ‘truncate’ values that exceed *t*^∗^ by setting  Then RMST = is the usual definition of a mean applied to the truncated survival times. With right censoring, an alternative formula is needed, namely RMST =, where *S*(*t*) is an estimate of the survival function for the *n* patients [8].

**Figure 1:**
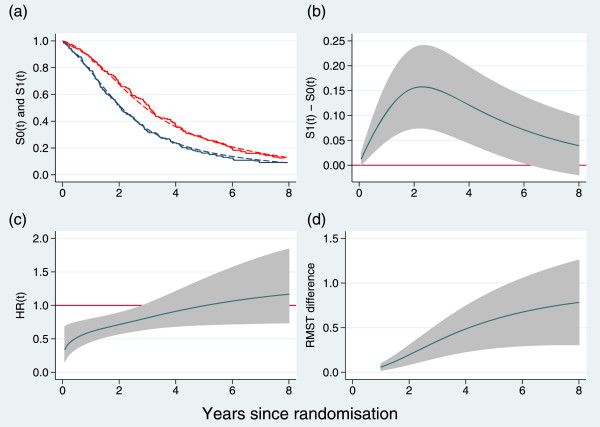
**Time-dependent outcome measures exemplified by the GOG111 trial in advanced ovarian cancer.** **(a)** Kaplan-Meier curves (solid lines) and estimated survival functions (dashed lines) from a flexible parametric model; **(b)** difference in survival functions; **(c)** instantaneous hazard ratio; **(d)** difference in restricted mean survival time. Shaded areas are pointwise 95% confidence intervals. Estimates in panels **(b)**, **(c)** and **(d)** are derived from a flexible parametric model.

An example of a graphic illustrating the four outcomes for the GOG111 trial in ovarian cancer [9] is shown in Figure 1. The estimates are plotted on a continuous time-scale to avoid imposing arbitrary cut-points. We have truncated the estimates in Figure 1 to 8 years’ follow-up since there is almost no data beyond this point. Having sufficient follow-up is critical to obtaining reliable clinical and statistical assessment of a treatment’s effectiveness but is rarely emphasized in trial reports.

Figure 1(c) indicates that the treatment effect on the relative hazard scale is largest near *t*=0 and dwindles to HR =1 by about *t*=4 yr. The survival curves do not cross within the interval (0,8) yr, so the RMST difference, which is the integrated difference between the survival curves, increases in a monotone fashion. By *t*=8 yr it reaches about 0.8 yr, albeit with a wide confidence interval.

#### Quantitative results

We propose that the primary quantitative estimates are taken as the overall HR with its confidence interval (noting that the instantaneous HR may be time-dependent) and the difference(s) in restricted mean survival time, with confidence interval(s).

The report of quantitative results should include the *P*-values from each of the three tests (the joint test and its two components), the joint test being the primary comparison and the two components as supporting evidence. The HR from a Cox model and results from Figure 1 at one to three clinically relevant time-point(s) should also be reported.

For advanced ovarian cancer, for example, reporting estimates at 2 and 5 years after randomization might be appropriate. The overall hazard ratio and the test results for the GOG111 trial are shown in the first row of Table 1. The joint test statistic is highly significant (*P*=0.0004), more so in fact than either of its components.

**Table 1:** **Overall hazard ratio and test statistics for the GOG111 and PATCH trials**

Trial	Hazard ratio		Joint test		Cox test of HR = 1		G-T test of PH	
	Est.	95% CI	Chi-sq.	***P***	Chi-sq.	***P***	Chi-sq.	***P***
GOG111	0.73	(0.59,0.90)	15.90	0.0004	8.38	0.0038	7.52	0.0061
PATCH1	0.71	(0.50,1.00)	7.58	0.023	3.78	0.052	3.80	0.051

Est. = estimate, Chi-sq. = chi-square.

Estimates of key quantities at 2 and 5 years of follow-up are given in Table 2. There is a fairly substantial treatment effect. For example, the RMST difference is ≥10*%* of the reference follow-up time *t*^∗^ at both 2 and 5 yr.

**Table 2:** **Time-dependent quantitative results for the GOG111 trial**

Quantity	***t***=2 yr		***t***=5 yr	
	Est.	95% CI	Est.	95% CI
HR (*t*)	0.71	(0.57,0.89)	0.99	(0.71,1.39)
*S*_0_(*t*)	0.52	(0.46,0.58)	0.18	(0.13,0.23)
*S*_1_(*t*)	0.68	(0.62,0.73)	0.27	(0.22,0.33)
*S*_1_(*t*)-*S*_0_(*t*)	0.16	(0.07,0.24)	0.09	(0.02,0.16)
RMST _0_ (yr)	1.53	(1.45,1.61)	2.44	(2.22,2.67)
RMST _1_ (yr)	1.73	(1.66,1.79)	3.04	(2.81,3.26)
RMST _1_- RMST _0_	0.20	(0.09,0.30)	0.59	(0.27,0.91)

Note that the estimated hazard ratio, HR(*t*), is instantaneous. See text for further details.

#### A second example

PATCH1 is a randomized, double-blind trial comparing a 12-month course of low-dose penicillin with a placebo in the prevention of recurrent cellulitis of the leg, a common baterial infection of the skin and underlying tissue [10]. The trial is rather small, with 129 events (patients experiencing one or more recurrences) among 274 patients followed up for a maximum of 3 years.

**Figure 2:**
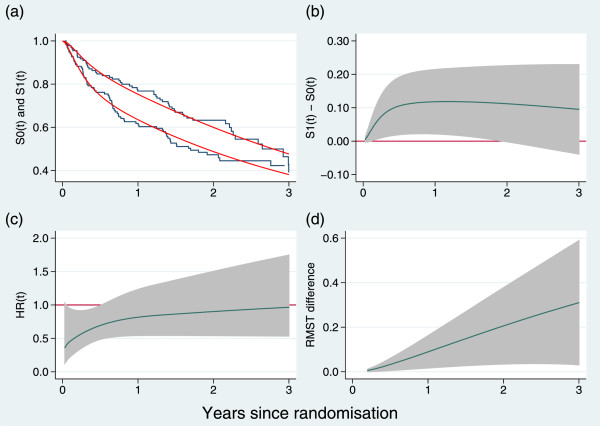
**Time-dependent outcome measures exemplified by the PATCH1 trial: (a) Kaplan- Meier curves (solid lines) and estimated survival functions (dashed lines) from a flexible parametric model; (b) difference in survival functions; (c) instantaneous hazard ratio; (d) difference in restricted mean survival time.** Shaded areas are pointwise 95 intervals. Estimates in panels **(b)**, **(c)** and **(d)** are derived from a flexible parametric model. Follow-up has been truncated at 3 years.

The results for PATCH1 are shown graphically in Figure 2 and in tabular form in Table 1 (we have omitted the equivalent of Table 2 since the results are indicated in Table 1 and Figure 2).

The results here are particularly intriguing. Neither of the individual treatment-effect tests are formally significant at the 5*%* level, although both are very close to significant (see Table 1). In contrast, the joint test has a *P*-value of 0.02. The conclusions from the trial analysis could hinge on which approach to testing was taken. Figure 2(c) shows that, as with GOG111, the treatment-effect HR appears substantial near *t*=0 and diminishes rapidly over the course of follow-up, being near 1 after 1 year. Note the generally large uncertainty in the estimates.

### Estimation

#### Model for a time-dependent treatment effect

In the preceding section, we have recommended a particular presentation of results without indicating how the results were obtained. Obviously, the Kaplan-Meier curves in Figure 1(a) and the hazard ratio estimate and tests in Table 1 are standard and familiar. This cannot be said of the other graphs and corresponding quantities in Table 2.

There are many ways in which the curves in Figure 1 could have been estimated. None can be said to be ‘standard’. Figure 1 was actually produced using a time-dependent, hazards-scaled flexible parametric survival model (FPM) [4, 5]. This is the model class we propose and focus on.

Let *x* denote the (binary) treatment indicator variable, coded 0 for the control arm and 1 for the research arm. In the FPM, the baseline log *cumulative* hazard function, ln*H*_0_(*t*)= ln*H*(ln*t*;*x*=0), is modelled parametrically as a restricted cubic spline function of ln*t*, say ln*H*_0_(*t*)=*s*_0_(ln*t*). The FPM we fitted can be written as
3

The function *θ*(*t*)=*θ*_0_+*θ*_1_ ln(*t*) describes the time-dependent behaviour of the ratio of log cumulative hazard functions between trial arms as a linear function of log time. This choice is similar to, but differs slightly from, Cox’s suggestion [11] for modelling a time-dependent hazard ratio. In the latter, the log hazard function is modelled as ln*h*(*t*;*x*)= ln*h*_0_(*t*)+*θ*(*t*)*x*= ln*h*_0_(*t*)+[*θ*_0_+*θ*_1_*f*(*t*)]*x*, where *f*(*t*) is some simple parametric function of time such as ln*t* or .

When *θ*(*t*) is constant, that is, when *θ*_1_=0, the cumulative hazards and hence also the hazards in the FPM are proportional between treatment groups. Equation (3) is then a PH model with HR equal to exp(*θ*_0_).

#### Predefining the statistical analysis

These days, randomized controlled trials are heavily regulated in various ways. One requirement is for a detailed statistical analysis plan to be drawn up before the trial data are finalized and analysed. The plan should lay out how the data will be analysed. The aim is to avoid any temptation on the part of the investigators to interpret the data over-optimistically by a data-driven selection of techniques and their results. In our context, we need to pre-specify the significance tests(s) that will be applied to gauge the evidence for a non-null treatment effect, and the model(s) that will be fitted to the data, from which key estimates will be derived.

The joint test is straightforward to specify since it requires the logrank or Cox chi-square value and the Grambsch-Therneau chi-square statistic using, for example, the rank transformation of the failure times. (Other time transformations are sometimes used, but no evidence that we are aware of favours any particular one.) The primary test of the treatment effect is then the sum of the two chi-squares, which is tested on 2 d.f. at the appropriate significance level. The overall HR estimate and its confidence interval are taken from the Cox regression.

Regarding the choice of FPM, we remarked that ‘a sensible default strategy … is to assign 3 d.f. to the baseline distribution and 1 d.f. to a time-dependent treatment effect to account for possible non-PH’ [2]. Further experience since that publication has confirmed that this model specification is flexible enough to fit the vast majority of trials we have encountered. In our approach, 3 d.f. means that the baseline *s*_0_(ln*t*) is a restricted cubic spline function with a boundary knot at each of the extreme failure times and interior knots at the 33rd and 67th centiles of the failure times. The baseline function is required in the estimation of the survival and other curves shown in Figures 1 and 2.

We have occasionally encountered trials in which the time-dependent component, *θ*_1_ ln(*t*), of the treatment effect is more complex than a logarithmic function of time. Such additional complexity could be investigated in secondary analyses. However, we judge that the simplicity and particularly the parsimony of model (3) are essential to a robust, pre-specified primary analysis. Similarly, 3 d.f. for the baseline spline may occasionally seem insufficient according to, say, an information criterion. Nevertheless, the improvement in fit to survival probabilities when further knots are included is typically small and of little practical importance. See also Royston and Lambert [5] pp. 74-79.

## Results

### Simulation study

We performed a small simulation study of the power of the joint test under non-PH. (The power under PH is known.) We powered a hypothetical trial design under the logrank test with a sufficient sample size and number of events for the joint test to have power 0.8 under PH, at a two-sided significance level of 0.05. The corresponding power of the logrank test under PH was 0.874. The target HR was taken to be 0.75. We assumed staggered entry of patients into the ‘trial’ at a uniform rate for 8 years, with a subsequent follow-up period of 4 years. Thus, the minimum and maximum possible follow-up times for any patient were 4 and 12 years, respectively. The required numbers of patients and events were 700 and 467, respectively.

**Figure 3:**
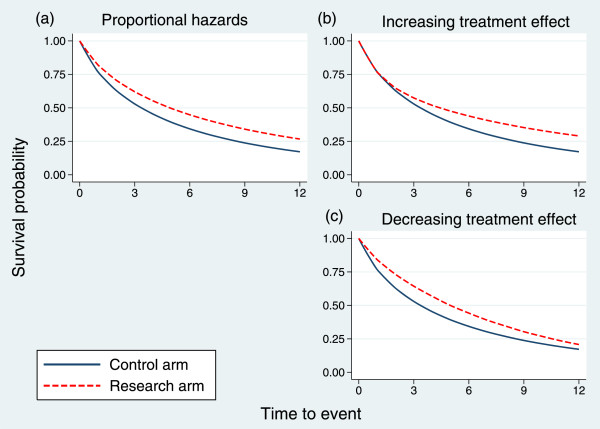
**Survival curves in the control and research arms for three hazard ratio patterns.** **(a)** Proportional hazards; **(b)** increasing treatment effect; **(c)** decreasing treatment effect.

Times to event were simulated under piecewise exponential models according to two patterns of non-proportional hazards, HR _1_(*t*) and HR _2_(*t*), representing increasing or decreasing treatment effects, respectively. The relevant design parameters are shown in Table 3. For example, *S*_0_(*t*)=0.767 at *t*=1 yr means that the control arm survival probability is 0.767 at *t*=1. The HR for the increasing treatment effect (HR _1_) is 1.00 in the interval *t*∈(0,1), and so the survival probability in the research arm would also be 0.767 at *t*=1. The pattern of the survival curves for PH and for the two non-PH cases is shown in Figure 3.

**Table 3:** **Design parameters for the simulation study of power**

Time,***t***	***S***_0_( ***t***)	HR _1_( ***t***)	HR _2_( ***t***)	Time,***t***	***S***_0_( ***t***)	HR _1_( ***t***)	HR _2_( ***t***)
1	0.767	1.00	0.65	7	0.302	0.60	1.0
2	0.628	0.85	0.70	8	0.268	0.60	1.0
3	0.529	0.70	0.75	9	0.238	0.60	1.1
4	0.453	0.65	0.80	10	0.213	0.60	1.1
5	0.392	0.60	0.90	11	0.191	0.60	1.2
6	0.343	0.60	0.90	12	0.172	0.60	1.2

The simulation was performed with 5,000 replicates. The results are shown in Table 4. Under both patterns of non-PH, the joint test outperforms the logrank test, markedly so when the treatment effect increases with time. Under PH, the power of the joint test is of course lower.

**Table 4:** **Power of tests under different scenarios: simulation results**

Test	Treatment effect		
	Constant (PH) ^***a***^	Increasing (HR _1_) ^***b***^	Decreasing (HR _2_) ^***c***^
Logrank	0.874	0.719 (0.006)	0.820 (0.005)
Joint	0.800	0.854 (0.005)	0.855 (0.005)

^*a*^Under proportional hazards with HR = 0.75.

^*b*^Simulation mean HR = 0.790.

^*c*^Simulation mean HR = 0.767.

Values under PH are exact theoretical figures. Other values are the proportions of 5,000 simulation replicates in which the joint null hypothesis was rejected. Values in parentheses are Monte Carlo standard errors.

### Numerical examples of sample size

To exemplify sample size and number of events under the joint test, we use the same design parameters as for the simulation study (see Table 3). We take the power for the joint test to be 0.709 or 0.835 (corresponding to power 0.8 or 0.9 for the logrank test - strategy 1) or 0.8 or 0.9 (typical power values chosen by practitioners - strategy 2). We set the target HR under PH to be 0.7, 0.75 or 0.8, values commonly encountered in real trials. The results are shown in Table 5.

**Table 5:** **Example of sample size calculation for the joint test under PH**

Power of test under PH		Target HR	Sample size	
Logrank	Joint		***n***	events
0.8	0.709	0.70	378	248
		0.75	570	380
		0.80	931	632
0.9	0.835	0.70	506	332
		0.75	763	509
		0.80	1246	845
0.874	0.8	0.70	464	304
		0.75	700	467
		0.80	1142	775
0.945	0.9	0.70	610	399
		0.75	919	613
		0.80	1500	1018

The power for the logrank test is given in the first column. The corresponding power of the joint test is shown in the second column.

In general, about 20 percent more events are required by the joint test than the logrank test. For example, for target HR =0.75, the joint test with power 0.9 requires logrank power 0.945 and 20.4 percent more patients and events than the logrank test with power 0.9 (919 versus 763 patients, 613 versus 509 events).

## Discussion

Our paper has two components: trial design with associated significance testing, and estimation of results. We discuss these briefly in turn.

There is no particular reason that we are aware of for expecting proportional hazards of the treatment effect. It is a convenient assumption that facilitates sample size calculation in time-to-event data. Our basic design idea is to improve the power to detect a more general, that is, potentially more complex, treatment effect than PH. The motivation is the increasingly frequent occurrence of non-PH in trials, with a concern that the power of the logrank test may be low in some of these cases. The outcome could be a trial declared or regarded as ‘negative’, when in fact a clinically relevant difference in survival curves between treatments was present.

There are costs to generalizing the concept of a treatment effect. Patterns of non-PH are potentially very varied, and it is hard, if not impossible, to design a trial with a convincing prior assumption about the likely pattern. Our proposed solution is to power the trial under PH according to a two-part (‘joint’) test. By combining the usual logrank or Cox test with the Grambsch-Therneau test of non-PH, we incur a loss of power under PH, but we may gain power under non-PH.

We discussed three possible strategies for trial design: (1) power according to the logrank test, with a hit in power of the joint test; (2) power according to the joint test, with an increase in sample size required via the higher power used in the logrank test; or (3) the same strategy as (2), but relaxing the significance level of the joint test to achieve the same power as the logrank test.

We have a slight preference for strategy 2. A frequent choice, for example, in past Medical Research Council (MRC) cancer trials has been to power the logrank test at 90 percent with significance level 5 percent and a target HR of 0.75. As we have seen, such a design guarantees power of 83.5 percent for the joint test under PH, which many would consider adequate. Others may have different preferences. We indicate how to do the relatively straightforward power calculations in the present paper. Sophisticated methodology and software (for example, [12, 13]) are available for implementing complex trial designs under the logrank test. These can of course also be used with the joint test under PH.

The Grambsch-Therneau test is based on scaled Schoenfeld residuals derived from a Cox PH model. Schoenfeld residuals are unsuitable for estimation of the quantities of substantive interest in a survival analysis of trial data. For that reason, for estimation, we suggest using a flexible parametric model with a time-dependent treatment effect. This class of models can be pre-specified in sufficient detail in a protocol and statistical analysis plan. It provides smooth estimates of survival probabilities, hazard ratio functions, restricted mean survival times, and so on. While there is a potential risk of bias due to the FPM failing to fit the data adequately, our experience so far is that noticeable lack of fit to the survival functions is uncommon. Of course, the Cox model can also fit badly.

The time-dependent treatment effect function incorporated in the FPM is log-linear in the follow-up time and therefore of limited flexibility. The fit can be checked by inspecting a plot of smoothed Schoenfeld residuals against the failure times, which gives a ‘non-parametric’ impression of the pattern of the log hazard ratio over time. If necessary, in secondary analysis the FPM can be elaborated with further spline parameters to improve the fit.

A sensible alternative to the joint test we describe is a joint test of the two parameters *θ*_0_ and *θ*_1_ in the FPM. This test, also on 2 d.f., is of the treatment effect and its interaction with (log) time. The global null hypothesis is *θ*_0_=*θ*_1_=0 (see Equation (3)). In an informal comparison using a database of 25 heterogeneous RCTs, we found good agreement and no consistent differences in the *P*-values of the two joint tests (data not shown). At this point, we have no empirical evidence to support recommending one test over the other. However, one theoretical consideration favouring the Cox/Grambsch-Therneau joint test is that the Grambsch-Therneau test is more general than the time-dependent function *θ*_0_+*θ*_1_ ln*t*. It is conceivable, therefore, that the Cox/Grambsch-Therneau test may tend to have higher power in general than the FPM-based joint test. On the other hand, some researchers may favour congruence between the global test for a treatment effect being based on the FPM and the same FPM being used in the description and interpretation of the trial results.

A key feature of the joint test is that it is sensitive to simple and also to more ‘complex’ treatment effects. In the latter case, assuming the result is not a type 1 error, the test is indicating there is a genuine difference between the survival curves. Even if the overall treatment effect, considered over the entire follow-up time of the trial, is small, the difference between the arms may still be of clinical and/or scientific interest and importance. For example, the difference in the survival curves between the treatment arms may suggest possible mechanisms of action of the treatments.

We are not suggesting that the joint test be adopted routinely. Primarily, we suggest that the trialist choose the preferred test according to the perceived modes of action of the treatments being compared. If the modes are obviously different, for example surgery versus a more conservative approach such as watchful waiting or a non-surgical therapy, the hazard functions will probably differ markedly in shape and non-PH seems more likely. The joint test may then be a good choice. If rather similar treatments are involved, such as various chemotherapy regimens, non-PH may seem less likely and the logrank test may be best. There may be indications of the extent and nature of non-PH from earlier trials or, in cancer for example, from other cancer types in which the treatment has been evaluated. Another consideration is judging how close to PH the ensuing survival curves are likely to be. If a treatment effect is expected to emerge relatively soon after randomization, non-PH is likely to be mild and the logrank test will be the more powerful. If the effect emerges much later in follow-up, the joint test is likely to be more powerful.

## Conclusion

The design and analysis of trials in the era of non-proportional hazards needs to accommodate a wider consideration of the nature of treatment effects. We have suggested one way forward which retains the tools familiar in the standard approach to trial design and sample size under proportional hazards, but which utilizes a joint test of the null hypothesis with power against non-proportional hazards. The trialist can choose whether to pay the price of the generalization by increasing the sample size or relaxing the significance level used in the joint test. We recommend the former. For analysis of the trial data, our approach pre-specifies a flexible parametric model that can represent a time-dependent hazard ratio if one is present.

## Authors’ information

Both authors are biostatisticians. MKBP is the director of the MRC Clinical Trials Unit at UCL. PR is a senior statistician in the same unit and an honorary professor of statistics at UCL.

## Abbreviations

CI: confidence interval
d.f.: degrees of freedom
FPM: flexible parametric model
HR: hazard ratio
MRC: Medical Research Council
non-PH: non-proportional hazards
PH: proportional hazards
RCT: randomized controlled trial
RMST: restricted mean survival time.
